# Multilocus sequence typing and *ftsI* sequencing: a powerful tool for surveillance of penicillin-binding protein 3-mediated beta-lactam resistance in nontypeable *Haemophilus influenzae*

**DOI:** 10.1186/1471-2180-14-131

**Published:** 2014-05-20

**Authors:** Dagfinn Skaare, Inger Lill Anthonisen, Dominique A Caugant, Andrew Jenkins, Martin Steinbakk, Linda Strand, Arnfinn Sundsfjord, Yngvar Tveten, Bjørn-Erik Kristiansen

**Affiliations:** 1 Department of Microbiology, Vestfold Hospital Trust, Tønsberg, Norway; 2 University of Tromsø, Tromsø, Norway; 3 Department of Bacteriology and Immunology, Norwegian Institute of Public Health, Oslo, Norway; 4 Faculty of Medicine, University of Oslo, Oslo, Norway; 5 Department of Environmental and Health Sciences, Telemark University College, Bø, Norway; 6 Telemark Hospital, Skien, Norway

**Keywords:** *Haemophilus influenzae*, Beta-lactam resistance, *ftsI*, PBP3, BLNAR, MLST, PFGE, Horizontal gene transfer, Recombination, Surveillance

## Abstract

**Background:**

Beta-lactam resistance in *Haemophilus influenzae* due to *ftsI* mutations causing altered penicillin-binding protein 3 (PBP3) is increasing worldwide. Low-level resistant isolates with the N526K substitution (group II low-rPBP3) predominate in most geographical regions, while high-level resistant isolates with the additional S385T substitution (group III high-rPBP3) are common in Japan and South Korea.

Knowledge about the molecular epidemiology of rPBP3 strains is limited. We combined multilocus sequence typing (MLST) and *ftsI*/PBP3 typing to study the emergence and spread of rPBP3 in nontypeable *H. influenzae* (NTHi) in Norway.

**Results:**

The prevalence of rPBP3 in a population of 795 eye, ear and respiratory isolates (99% NTHi) from 2007 was 15%. The prevalence of clinical PBP3-mediated resistance to ampicillin was 9%, compared to 2.5% three years earlier. Group II low-rPBP3 predominated (96%), with significant proportions of isolates non-susceptible to cefotaxime (6%) and meropenem (20%). Group III high-rPBP3 was identified for the first time in Northern Europe.

Four MLST sequence types (ST) with characteristic, highly diverging *ftsI* alleles accounted for 61% of the rPBP3 isolates. The most prevalent substitution pattern (PBP3 type A) was present in 41% of rPBP3 isolates, mainly carried by ST367 and ST14. Several unrelated STs possessed identical copies of the *ftsI* allele encoding PBP3 type A.

Infection sites, age groups, hospitalization rates and rPBP3 frequencies differed between STs and phylogenetic groups.

**Conclusions:**

This study is the first to link *ftsI* alleles to STs in *H. influenzae*. The results indicate that horizontal gene transfer contributes to the emergence of rPBP3 by phylogeny restricted transformation.

Clonally related virulent rPBP3 strains are widely disseminated and high-level resistant isolates emerge in new geographical regions, threatening current empiric antibiotic treatment. The need of continuous monitoring of beta-lactam susceptibility and a global system for molecular surveillance of rPBP3 strains is underlined. Combining MLST and *ftsI*/PBP3 typing is a powerful tool for this purpose.

## Background

*Haemophilus influenzae* is a major cause of respiratory tract infections and invasive disease, with encapsulated strains of serotype b (Hib) being most virulent [1]. Nontypeable isolates (NTHi) now account for the majority of cases of invasive disease in countries where Hib conjugate vaccines have been introduced [2-4]. NTHi vaccines have a huge potential for further reducing the global burden of disease but are not yet available [1,5].

Beta-lactams are first-line drugs for treatment of *H. influenzae* infections but resistance may develop due to transferable beta-lactamases (impacting penicillins only) or alterations in the transpeptidase domain of penicillin-binding protein 3 (PBP3), encoded by the *ftsI* gene (impacting all beta-lactams) [6]. Traditionally, isolates with the latter resistance mechanism have been denoted beta-lactamase negative ampicillin resistant (BLNAR), whereas isolates with both mechanisms have been denoted beta-lactamase positive amoxicillin-clavulanate resistant (BLPACR). PBP3-mediated resistance is defined by the presence of particular amino acid substitutions (Table 1): R517H or N526K near the KTG motif in low-level resistant isolates (groups I and II, respectively), and the additional substitution S385T near the SSN motif in high-level resistant isolates (group III-like, S385T + R517H; group III, S385T + N526K) [7-10].

**Table 1:** **Genotypes of PBP3-mediated resistance in *Haemophilus influenzae***

**Genotype designations**^**a**^			**PBP3 substitutions**^**b**^		
			**SSN**	**KTG**	
**Category**^**c**^	**Level**	**Group**	**S385**	**R517**	**N526**
rPBP3	High	III^d^	T		K
		III-like^e^	T	H	
	Low	II			K
		I		H	
sPBP3	NA	NA			

^a^According to Ubukata *et al.*[7], Hasegawa *et al.*[8], Garcia-Cobos *et al.*[9], Hotomi *et al.*[10] and this study. NA, not applicable.

^b^Essential amino acid substitutions in PBP3 (transpeptidase domain, 338–573) with the amino acid sequence of *H. influenzae* Rd KW20 [GenBank:U32793] as reference. SSN, Ser-Ser-Asn motif; KTG, Lys-Thr-Gly motif.

^c^rPBP3, isolates with PBP3 sequences conferring resistance to beta-lactams (isolates assigned to groups I, II, III-like and III); sPBP3, isolates with PBP3 sequences conferring wild-type susceptibility to beta-lactams (remaining isolates).

^d^Originally reserved for isolates with the additional substitutions M377I and L389F by Ubukata *et al.*[7], modification proposed by Hotomi *et al.*[10].

^e^Originally categorized as group I by Ubukata *et al.*[7], new group assignment proposed by Garcia-Cobos *et al.*[9].

An increased prevalence of PBP3-mediated resistance (hereafter denoted rPBP3) has been observed worldwide [2,4,11-16]. Isolates with high-level resistance (high-rPBP3) are a major clinical problem in Japan and South Korea [15-17] whereas low-level resistant (low-rPBP3) isolates so far predominate in the rest of the world [4,11,12,14,18-21]. Group II isolates with a characteristic substitution pattern, PBP3 type A (D350N, M377I, A502V, N526K, V547I and N569S) [11], and compatible patterns (identical to PBP3 type A as far as comparison is possible) are particularly common [3,4,9,11,12,16,18,20],[22-25]. The mechanisms by which rPBP3 isolates emerge are not fully understood. Spontaneous mutations are considered the primary cause of the substitutions R517H, N526K and S385T [6,26] but horizontal gene transfer (HGT) by classical transformation and homologous recombination has been suggested to play an important role in the further development and spread of resistance [11,26-28].

Clonal spread of rPBP3-NTHi is extensively documented [3,4,6,9-11,18,26]. However, knowledge about the molecular epidemiology of rPBP3 strains is limited. Previous studies based on pulsed-field gel electrophoresis (PFGE) and other molecular methods have generated results not easily compared between studies. Multilocus sequence typing (MLST) has the advantage of providing objective, unambiguous data, easy to compare and well suited for assessment of phylogenetic relationship in both encapsulated isolates and NTHi [29,30]. The MLST scheme for *H. influenzae* assigns isolates to sequence types (ST) based on allelic profiles of the seven housekeeping genes *adk*, *atpG*, *frdB*, *fucK*, *mdh*, *pgi* and *recA*[30]. Software for phylogenetic analysis and a continuously updated database with STs, serotypes and clinical data (but not resistance genotypes) is available on the website http://haemophilus.mlst.net. MLST has improved our understanding of population structure in *H. influenzae*[29-32]. A maximum-parsimony analysis of concatenated sequences from all isolates in the database has identified 14 phylogenetic groups (Clades 1–13 and eBURST group 2) with different genetic characteristics, including serotypes and virulence determinants [32].

The objectives of this study were to: 1) Estimate the prevalence of rPBP3 in eye, ear and respiratory isolates of *H. influenzae* in Norway and map PBP3 genotypes and phenotypic beta-lactam susceptibility profiles; 2) Examine the molecular epidemiology of rPBP3 isolates and seek for evidence of HGT; and 3) Explore any associations between phylogeny, resistance genotypes and pathogenicity, as reflected by clinical characteristics (age, gender, hospitalization rates and sample types).

## Methods

### Bacterial isolates

**Figure 1:**
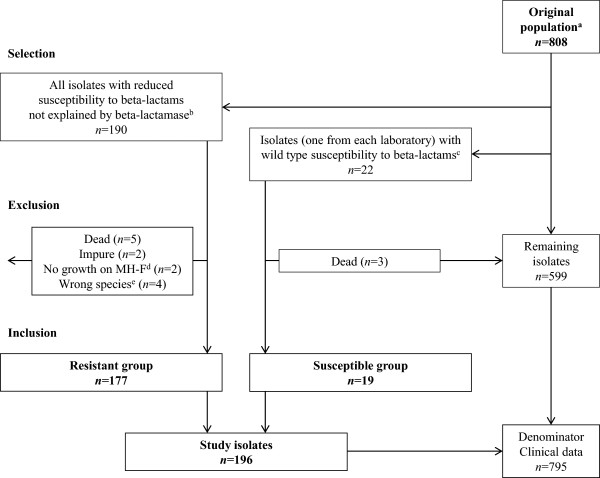
**Study isolates.** Flowchart showing selection and inclusion of bacterial isolates. ^a^NORM 2007 surveillance population [33]. ^b^According to phenotypic susceptibility profiles (by gradient MIC, disk diffusion and beta-lactamase detection) as reported by the primary laboratories. The following selection criteria were used: amoxicillin-clavulanate MIC ≥2 mg/L, cefuroxime MIC ≥4 mg/L, cefotaxime MIC ≥0.12 mg/L and/or cefaclor 30 μg zone <17 mm (all isolates); and ampicillin MIC ≥1 mg/L, phenoxymethylpenicillin 10 μg zone <13 mm and/or ampicillin 2 μg zone <16 mm (beta-lactamase negative isolates). The selection criteria were constructed using epidemiological cut-off MIC values defined by EUCAST (http://www.eucast.org/MIC_distributions) and zone diameter distributions from the surveillance report [33]. Information about the methodologies for susceptibility testing are included in the surveillance report [33]. ^c^One beta-lactamase negative isolate from each laboratory, randomly selected from the isolates remaining after selection for the Resistant group. ^d^MH-F, Mueller-Hinton agar supplemented with defibrinated horse blood and β-NAD for susceptibility testing of fastidious organisms (http://www.eucast.org). ^e^*H. parainfluenzae* (*n =* 3) and *H. haemolyticus* (*n =* 1).

One hundred and seventy-seven *H. influenzae* isolates with a phenotype suggesting rPBP3 (Resistant group, R-group) and 19 isolates with wild-type susceptibility to beta-lactams (Susceptible group, S-group) were characterized. The isolates were selected from a population of 808 consecutive eye, ear and respiratory tract isolates, collected as part of standard patient care in January and February 2007, included in the Norwegian Surveillance Programme for Antimicrobial Resistance (NORM) [33]. Selection was based on the susceptibility profiles reported by the primary laboratories (Figure 1). Thirteen isolates were selected but excluded for various reasons. Clinical information (site of isolation; age and gender of the patient; hospitalization status at the time of sampling) for the 196 study isolates and 599 isolates in the original population was used for statistical analyses.

PFGE band patterns and *ftsI* sequences for 46 *H. influenzae* isolates from a comparable population collected in 2004, characterized in a previous study [11], were included in the phylogenetic analyses.

### Species identification and serotyping

Isolates were inoculated on chocolate agar and incubated overnight at 35 ± 1°C in ambient air with 5% CO_2_. After control of purity and presumptive identification by smell, colony morphology and dependence of β-NAD and haemin, isolates were frozen at −70°C using Microbank vials (Pro-Lab Diagnostics, Richmond Hill, Ontario, Canada). Species identification was confirmed by outer membrane protein P6 (*ompP6*) and 16S rRNA PCR using primers as described previously [34] and probes designed for this study (Table 2). Where this test was negative (*n* = 10), a 547 bp fragment of the 16S rRNA gene was sequenced at GATC Biotech (Konstanz, Germany) to confirm species identification.

**Table 2:** New and modified primers and probes used in this study

**Name**	**Function**	**Target**	**Sequences (5′ to 3′)**^**a**^	**Original (reference)**
*bexA*Fb	F-primer	*bexA*	CGTTT**A**T**R**TGATGTTGATCC**T**GA	HI-1 [35]
*bexA*Rb	R-primer	*bexA*	TGTCCAT**A**TCTTCAAAATG**G**TG	HI-2 [35]
*bexA*P	Probe	*bexA*	FAM ATGCAAGYCGRGCTTTCATCCCTG-BHQ	This study
Hinf_fR	R-primer	*cap* (serotype f)	G**G**TACTATCAAGTCCAAATC	f3 [35]
Hinf_eR2	R-primer	*cap* (serotype e)	CTAATTGTTCTTTCTGTCTA	This study
*ompP6*P	Probe	*ompP6*	ACG TGG TAC ACC AGA ATA CAA CAT CGA	This study
H16SP	Probe	16S rRNA gene	TCGCTCCACCTCGCAGCTTCGCT	This study
TEMP	Probe	*bla*_TEM_	CAG CTC CGG TTC CCA ACG ATC AAG	This study
ROBP	Probe	*bla*_ROB_	TAG CGA CAA CAG CGC GAC CAA TTT G	This study

^a^Sites of modifications in bold.

Capsular serotyping was done by *bexA* PCR and capsule type-specific PCRs for *bexA* positive isolates as described previously [35], with modifications to the HI-1, HI-2 and f3 primers. A new serotype e-specific reverse primer and a *bexA* probe were designed for this study (Table 2).

### Susceptibility testing

MIC determination by microbroth dilution (HTM, Oxoid Ltd, Basingstoke, UK) was carried out according to CLSI guidelines [36], except that testing of penicillin-beta-lactamase inhibitor combinations was performed with fixed inhibitor concentrations [37]. Beta-lactam agents tested were ampicillin, amoxicillin, piperacillin, cefuroxime, cefotaxime (Sigma-Aldrich, St. Louis, MO, USA) and meropenem (Sequoia, Pangbourne, UK). For beta-lactamase positive isolates, ampicillin, amoxicillin and piperacillin MICs were determined in the presence of sulbactam 4 mg/L (Sequoia), clavulanate 2 mg/L and tazobactam 4 mg/L (Sigma-Aldrich), respectively. MICs were within accepted ranges for *H. influenzae* ATCC 49247 (rPBP3) and *H. influenzae* ATCC 49766 (sPBP3), and within the wild type range (http://www.eucast.org/MIC_distributions) for *H. influenzae* ATCC 35056 (TEM-1 positive).

MICs were interpreted according to EUCAST clinical breakpoints, except for piperacillin and piperacillin-tazobactam where breakpoints are not defined [37]. Meningitis breakpoints were used for susceptibility categorization of meropenem to allow quantification of low-level resistance. Data from this study are included in the EUCAST database for MIC distributions of clinical isolates.

### Resistance genotyping

PCR and sequencing of the transpeptidase domain of the *ftsI* gene were performed as described previously [11]. DNA sequences were analysed using Lasergene software (DNASTAR, Madison, WI, USA) and the sequences (nucleotides 1010–1719) have been deposited in the EMBL Nucleotide Sequence Database [EMBL:HG818627-818822].

**Figure 2:**
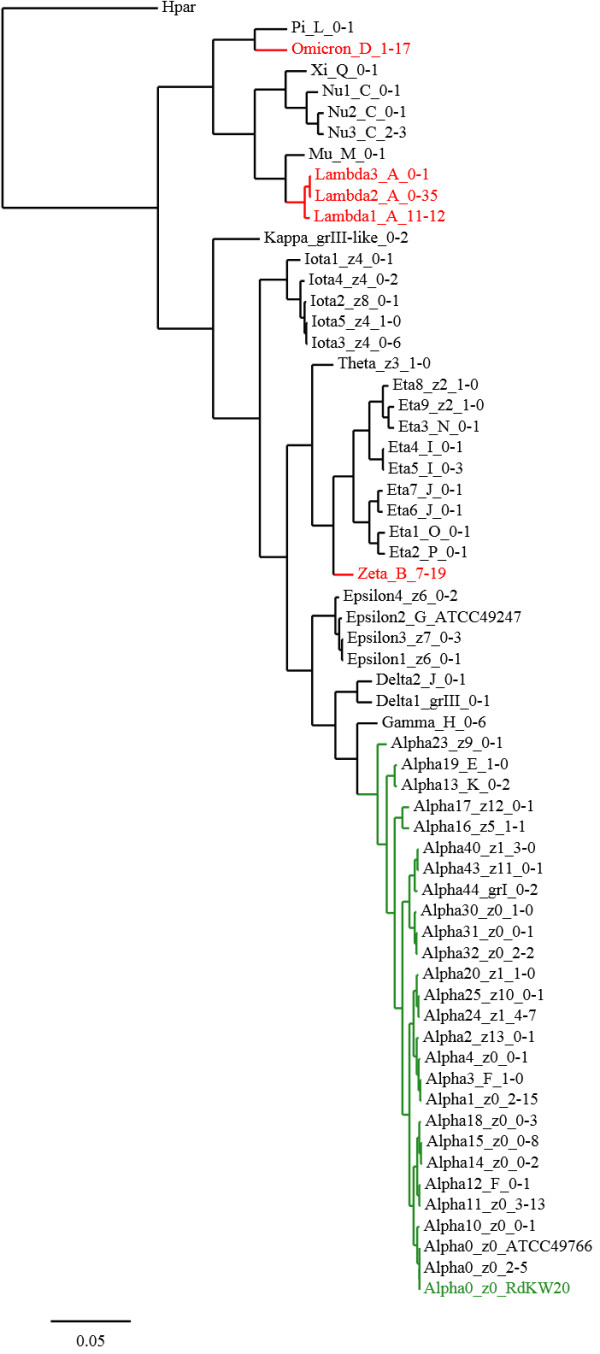
***ftsI *phylogram.** UPGMA phylogram of *ftsI* DNA sequences (transpeptidase domain, nucleotides 1010–1719) in the current (*n =* 196) and previous study (*n =* 46) [11]. The outgroup (Hpar) is *H. parainfluenzae* [EMBL:AB267856] and the reference sequence (z0) is *H. influenzae* Rd KW20 [GenBank:U32793]. The *H. influenzae* reference strains ATCC 49247 and ATCC 49766 are also included. The scale is DNA sequence divergence (0.05 = 5% divergence). Labels indicate *ftsI* alleles, PBP3 types and number of isolates with the particular allele in the previous and current study, respectively. The reference cluster *alpha* (green) and the alleles encoding PBP3 types A, B and D (red) are highlighted.

An UPGMA (unweighted pair group method with arithmetic mean) phylogram of *ftsI* alleles from this and a previous study [11] was constructed by distance methods using ClustalW2 (http://www.ebi.ac.uk) and displayed using TreeDyn software (http://www.phylogeny.fr) with *H. parainfluenzae* [EMBL:AB267856] as outgroup (Figure 2). Clusters of closely related alleles were assigned Greek letters (*alpha* – *pi*) with numbers denominating alleles within each cluster.

According to PBP3 substitution patterns (Table 1), isolates were categorized into resistance genotypes (Table 3). Group II rPBP3 isolates and isolates lacking essential substitutions (denoted sPBP3) were assigned to PBP3 types (A – Q and z1 – z13, respectively) according to the previously established system [11], further developed in this study.

**Table 3:** Resistance genotypes, PBP3 types and PBP3 substitutions

**Resistance genotypes**^**a**^	**PBP3 types**^**b**^	***n***^**c**^	**Sg**^**d**^	**Bla**^**e**^	**PBP3 substitutions**^**f**^																		
					**D**	**S**	**A**	**M**	**S**	**P**	**A**	**I**	**G**	**A**	**V**	**R**	**N**	**A**	**T**	**V**	**D**	**A**	**N**
					**350**	**357**	**368**	**377**	**385**	**392**	**437**	**449**	**490**	**502**	**511**	**517**	**526**	**530**	**532**	**547**	**551**	**554**	**569**
High-rPBP3																							
Group III	-	1			N	N			**T**					T			**K**^g^			I			S
Group III-like	-	2			N	N		I	**T**							**H**			S	I			
Low-rPBP3																							
Group II	A	48		1	N			I						V			**K**^h^			I			S
	B	19		5								V					**K**^g^			I			S
	C	5			N			I					E				**K**^h^			I			S
	D	17			N								E				**K**^g^	S					
	F	1															**K**^g^						
	H	6												V			**K**^h^						
	I	4			N						S			V			**K**^g^			I			S
	J	3			N									T			**K**^g^			I			S
	K	2					T							T			**K**^g^						
	L	1			N								E				**K**^g^			I		D^i^	S
	M	1			N									V			**K**^h^			I			S
	N	1		1	N						S	V					**K**^g^			I			S
	O	1												T			**K**^g^			I			S
	P	1												T			**K**^g^			I			
	Q	1											E	V			**K**^h^			I			S
Group I	-	2														**H**				I		T	
sPBP3	z0	51	15	6																			
	z4	9	1		N															I			S
	z1	7	3	2																I			
	z6	3																		I			S
	z7	3																		I		T	S
	z5	1		1										S									
	z8	1			N									T						I			S
	z9	1			N															I			
	z10	1																		I	A^i^		
	z11	1													A					I			
	z12	1								S^i^													
	z13	1												T									

^a^See Table 1.

^b^PBP3 types according to Skaare *et al.*[11] (types A – G) and this study (types H – Q and z0 – z13). ‘-‘, not designated.

^c^*n*, No. of study isolates.

^d^Sg, No. of isolates from the Susceptible group included in *n*.

^e^Bla, No. of beta-lactamase positive isolates (all TEM-1) included in *n*.

^f^Amino acid substitutions in PBP3 (transpeptidase domain, 338–573) with the amino acid sequence of *H. influenzae* Rd KW20 [GenBank:U32793] as reference (z0). Essential substitutions in bold.

^g^N526K encoded by the DNA triplet AAG.

^h^N526K encoded by the DNA triplet AAA.

^i^Novel substitution.

The DNA and PBP3 sequences of *H. influenzae* Rd KW20 [GenBank:U32793] were used as references (*alpha-0* and z0, respectively).

Isolates reported as beta-lactamase positive by the primary laboratory and isolates with a phenotype suggesting beta-lactamase production were examined by TEM-1 and ROB-1 PCR as described previously [38], with detection of PCR products by probes designed for this study (Table 2).

### Molecular strain characterization

**Figure 3:**
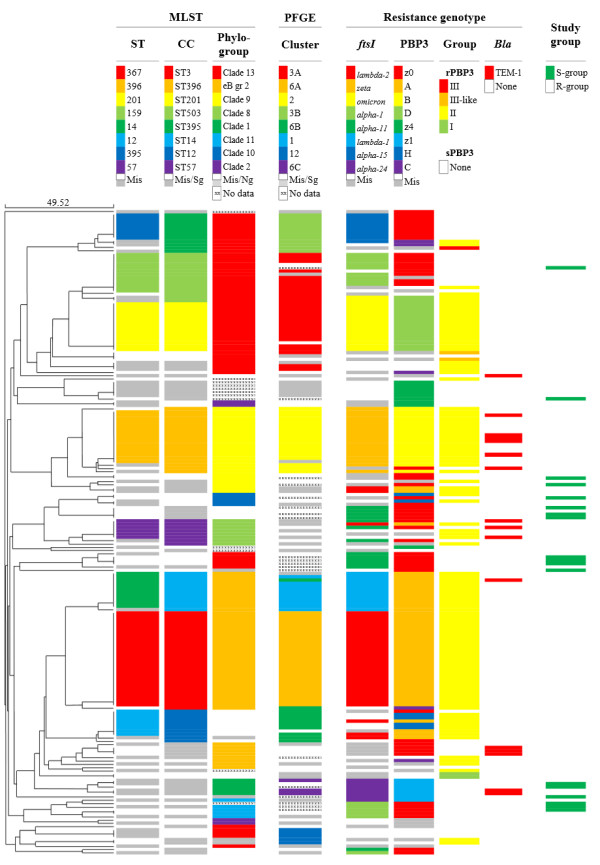
**MLST dendrogram.** The correlation between phylogenetic groups (MLST and PFGE) and resistance genotypes. UPGMA dendrogram of STs based on pair-wise differences in allelic profiles of the 196 study isolates with additional information about CCs, phylogroups, PFGE clusters, *ftsI* alleles, PBP3 types, PBP3 groups, beta-lactamase and study groups. The colour scale indicates relative frequencies of various alternatives within each of the columns 1–6. eB gr2, eBURST group 2; Mis, miscellaneous; Sg, singletons; Ng, no phylogroup; S-group, Susceptible group; R-group, Resistant group.

MLST was performed by standard procedures with sequencing of internal fragments of the seven housekeeping genes *adk*, *atpG*, *frdB*, *fucK*, *mdh*, *pgi* and *recA*[30]. Following registration of sequences at http://haemophilus.mlst.net for assignment of allele numbers and STs, data were analysed using software available on the website, with the construction of an UPGMA dendrogram based on the pairwise differences in allelic profiles (Figure 3), and division of STs into clonal complexes (CC) using eBURSTv3. The criterion for assignment to a CC (named according to the predicted founder) was sequence identity with another member of the complex at at least six loci [31].

STs were assigned to phylogenetic groups (here denoted phylogroups) according to previously performed maximum parsimony analysis of all STs in the MLST database [32]. More recent STs, not encompassed by the parsimony analysis, were indirectly assigned to phylogroups if they belonged to CCs encompassing STs with known phylogroup.

**Figure 4:**
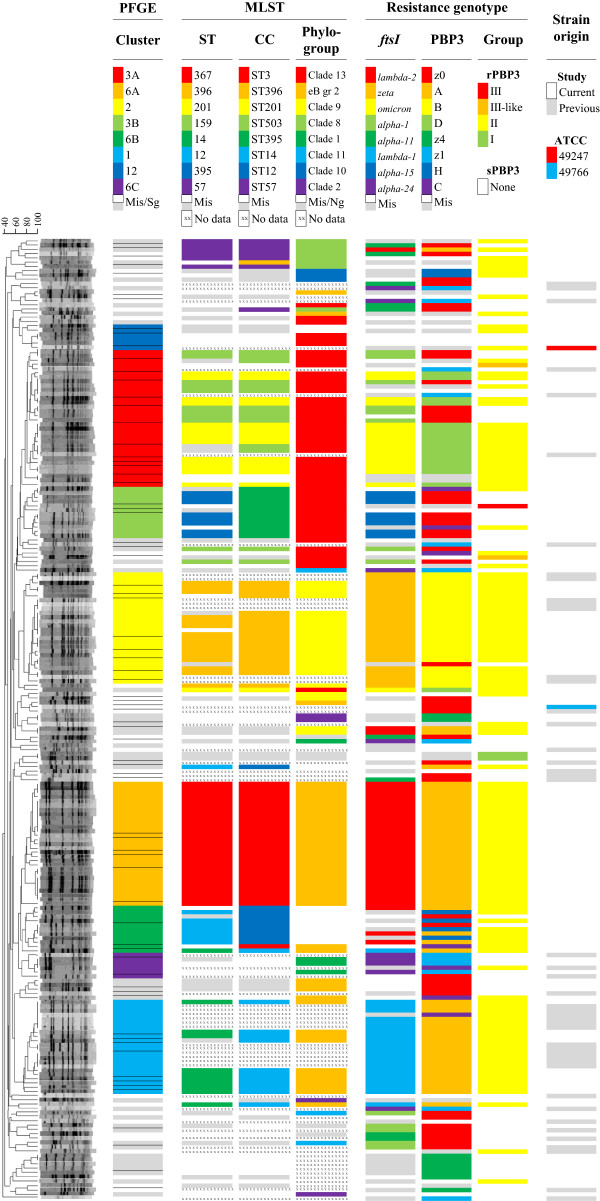
**PFGE dendrogram.** The correlation between phylogenetic groups (PFGE and MLST) and resistance genotypes. UPGMA dendrogram of band patterns for the 177 isolates in the Resistant group and 46 isolates from a previous study [11]. Clusters of related or possibly related isolates by analysis of band patterns and Dice coefficient of similarity are indicated by colours. Horizontal lines separate different band patterns. Additional information about STs, CCs, phylogroups, *ftsI* alleles, PBP3 types, PBP3 groups and strain origin is provided. The colour scale (similar to Figure 3) indicates relative frequencies of various alternatives within each of the columns 1–6. eB gr2, eBURST group 2; Mis, miscellaneous; Sg, singletons; Ng, no phylogroup.

PFGE of the 177 isolates in the R-group was carried out as described previously [11,38]. A dendrogram of band patterns, with 46 isolates from our previous study included [11], was constructed using GelCompare II software (Applied Maths, Sint-Martens-Latem, Belgium), Dice coefficients of similarity and the UPGMA algorithm (Figure 4). Clusters of related or possibly related isolates were identified by comparison of band patterns [39] and numbered according to the system established previously [11].

### Statistics

Multivariate regression analysis and Fisher’s exact test was performed using Predictive Analytics Software (PASW) Statistics version 17.0 (IBM Corporation, US).

### Ethics

The bacterial isolates and patient information used in this study were collected as part of the Norwegian Surveillance Programme for Antimicrobial Resistance (NORM). The NORM programme is warranted in Norwegian law (http://lovdata.no, FOR-2003-11-14-1353) and no further ethical approval was required for the use of isolates and data in this study.

## Results

### Resistance genotypes

In the R-group (n = 177), 116 isolates (66%) had essential PBP3 substitutions and were categorized as rPBP3. The remaining 61 isolates in the R-group, and all 19 isolates in the S-group, lacked essential substitutions and were categorized as sPBP3 (Table 4).

**Table 4:** **Frequencies of beta-lactam resistance and clinical characteristics in study groups and in the original population**^**a**^

		**rPBP3**^**c**^		***Bla***^**d**^		**Proportions (%) of isolates and patients**					
**Groups of isolates**^**b**^	***n***	***n***	**%**	***n***	**%**	**Anatomical sites**			**Age groups**		**Hospitalized**^**e**^
						**Eye**	**Ear**	**Respiratory**	**0-3**	**≥50**	
Resistant group	177	116	66	16	9	28	10	58	44	24	33
Susceptible group	19	0	0	0	0	21	32	42	68	5	11
Remaining isolates	599	0^f^	0^f^	60^g^	10^g^	19	15	63	41	22	23
Original population	795^h^	116	15	76	10	21	14	62	43	22	25

^a^NORM 2007 surveillance population [33], consisting of consecutive routine isolates from patients with eye, ear and respiratory tract infections.

^b^See text and Figure 1 for definition of the study groups (Resistant group and Susceptible group).

^c^PBP3-mediated resistance (see Table 1).

^d^Beta-lactamase positive.

^e^Proportions of patients hospitalized at the time of sampling.

^f^Assuming that all rPBP3 isolates were selected for the Resistant group.

^g^As reported by the primary laboratories.

^h^Thirteen isolates were selected for the Resistant group but excluded for various reasons (see Figure 1).

Most rPBP3 isolates were group II (111/116, 96%), including seven TEM-1 positive isolates, but one group III and two group III-like high-rPBP3 isolates were also identified (Table 3). The rPBP3 prevalence in the original population was thus 15% (116/795) and the prevalence of combined rPBP3 and TEM-1 was 0.9% (7/795).

Eighteen PBP3 substitution patterns were present in rPBP3 isolates, with PBP3 types A, B and D accounting for 72% (84/116) and PBP3 type A alone accounting for 41% (48/116). The N526K substitution was encoded by the DNA triplets AAA and AAG in 54% (61/112) and 46% (51/112) of the cases, respectively.

Analysis of the 49 *ftsI* alleles in the current study identified 14 clusters (Figure 2). PBP3 types A, B and D were confined to distinct clusters (*lambda*, *zeta* and *omicron*), all highly divergent from the reference sequence. Type A was encoded by three closely related alleles (cluster *lambda*) whereas types B (*zeta*) and D (*omicron*) showed no allelic diversity. Several clusters encompassed more than one PBP3 type, but only type J appeared in more than one cluster (*eta* and *delta*). The *lambda-1* and *zeta* alleles, encoding PBP3 types A and B, respectively, were highly prevalent in both sampling periods.

### Serotypes and phylogeny

Except for two serotype f (Hif) ear and respiratory tract isolates, all study isolates were nontypeable.

The 196 isolates represented 70 STs; hereunder 15 novel (ST1190 through ST1204, represented by one isolate each) (Figure 3). Eight STs had >5 representatives and accounted for 54% (105/196) of the isolates (Table 5). By eBURST analysis, the STs were grouped into 39 clonal complexes (CC) and three singletons.

**Table 5:** Frequencies of beta-lactam resistance and clinical characteristics of study isolates according to STs

		**rPBP3**^**a**^		***Bla***^**b**^		**Proportions (%) of isolates and patients**^**c**^					
**STs**	***n***	***n***	**%**	***n***	**%**	**Anatomical sites**			**Age groups**		**Hospitalized**^**d**^
						**Eye**	**Ear**	**Respiratory**	**0-3**	**≥50**	
ST367	29	29	100	0	0	17	17	59	28	34	28
ST396	16	16	100	5	31	**56**^**e**^	6	38	**81**^**f**^	13	38
ST201	15	15	100	0	0	**53**^**e**^	0	47	47	27	47
ST159	12	1	8	0	0	8	8	75	33	42	50
ST14	11	11	100	1	9	18	0	73	64	9	55
ST12	8	7	88	0	0	50	13	38	38	13	25
ST395	8	0	0	0	0	**63**^**e**^	0	25	63	25	0
ST57	6	4	67	3	50	33	17	50	83	17	33
Other STs	91	33	36	7	8	19	16	60	58	19	25
All STs	196	116	59	16	8	27	12	56	46	22	31

^a^PBP3-mediated resistance (see Table 1).

^b^Beta-lactamase positive (all TEM-1).

^c^Proportions for each ST were compared with the proportions for other STs (e.g. ST396 versus non-ST396) using Fisher’s exact test. Characteristics significantly more prevalent in particular STs are indicated (bold).

^d^Proportions of patients hospitalized at the time of sampling.

^e^p < 0.05.

^f^p = 0.004.

Direct assessment of phylogroup was possible for 32 STs (accounting for 129 isolates) and indirect assignment was possible for 30 STs (55 isolates). Eight STs (12 isolates) could not be assigned to a phylogroup. Ten out of 14 recognized phylogroups [32] were represented, and 69% of the isolates belonged to Clade 13 (*n =* 59), eBURST group 2 (*n =* 50) and Clade 9 (*n =* 26). The two Hif isolates (sPBP3, ST124) were in Clade 2.

The S-group was more diverse than the R-group and differed phylogenetically: fifteen STs were represented among 19 S-group isolates, with only one, ST159, being among the eight most frequent STs overall (Table 5). Two major R-group phylogroups (eBURST group 2 and Clade 8) were absent from the S-group.

Eight PFGE clusters of >5 isolates were identified, with Dice coefficients of clustering between 71% and 76% (Figure 4). PFGE clusters corresponded well to CCs, occasionally with a higher or lower resolution level. Additionally, two clusters (6B and 12) suggested genetic relationship (by three band difference) between isolates assigned to phylogroups (eBURST group 2 and Clade 13, respectively) and isolates with no phylogroup assignment, probably reflecting distant phylogenetic relationship not detected by the parsimony analysis.

### Phylogeny and resistance genotypes

The 116 rPBP3 and 80 sPBP3 isolates were distributed on 32 and 44 STs, respectively. Six of the 70 STs in this study (ST12, ST57, ST155, ST159, ST411 and ST422) included both categories. Most rPBP3 isolates (102/116, 88%) belonged to five phylogroups (rPBP3 proportions in brackets): eBURST group 2 (45/50, 90%); Clade 13 (28/59, 47%); Clade 9 (22/26, 85%); Clade 8 (5/8, 63%) or Clade 10 (2/4, 50%). The remaining 14 rPBP3 isolates lacked phylogroup assignment. The two group III-like and the single group III high-rPBP3 isolates were ST160 (no phylogroup) and ST1197 (Clade 13), respectively. No isolates in Clade 1 (*n* = 5), Clade 2 (*n* = 4), Clade 6 (*n* = 1), Clade 11 (*n* = 5) and Clade 12 (*n* = 2) were rPBP3.

The *ftsI* alleles *lambda-2*, *zeta* and *omicron*, encoding the three most frequent PBP3 types A, B and D, respectively, were, with a few notable exceptions, carried by ST367 (eBURST group 2), ST396 (Clade 9) and ST201 (Clade 13) (Figure 3). In addition, PBP3 type A encoded by the slightly different allele *lambda-1* was present in ST14, a triple locus variant of ST367 (both STs belong to eBURST group 2). These four strains (defined by combinations of STs and *ftsI* alleles) accounted for 61% (71/116) of the rPBP3 isolates in the current study.

Two strains frequently occurring in this study (ST14 with PBP3 type A and ST396 with PBP3 type B) had PFGE band patterns and *ftsI* alleles identical to strains in the two most prevalent resistant clones three years earlier (PFGE clusters 1 and 2, respectively) (Figure 4) [11].

Apart from ST367, PBP3 type A encoded by *lambda-2* was present in the following unrelated STs: ST57 (Clade 8), ST85 (Clade 9) and ST12 (no phylogroup). Similarly, the *ftsI* allele *gamma*, encoding PBP3 type H, was present in ST12 (no phylogroup) as well as the unrelated ST411 and ST422 (Clade 10). Conversely, seven STs hosted more than one PBP3 type. Notably, the six ST57 isolates carried four highly divergent rPBP3 types (A, K, L and N) and the reference sequence (z0).

Three ST57 isolates were TEM-1 positive but only one isolate had both TEM-1 and rPBP3. Most isolates with both resistance mechanisms (5/7, 71%) were ST396.

### Clinical characteristics

Clinical information for the 196 study isolates and the 599 remaining isolates in the original population is summarized in Table 4. For the study isolates, median age and age range of the patients were 5 (0 – 86) yrs with a male/female ratio of 1.0. The corresponding numbers in the original population were 5 (0 – 97) and 1.0.

Multivariate regression analysis of isolates with known hospitalization status (766/795, 96%) showed that increasing age (OR = 1.3, p < 0.001) and male gender (OR = 1.8, p = 0.001) were significant independent risk factors for hospitalization. With adjustment for age, gender and beta-lactamase production, there was a borderline significant association between rPBP3 and hospitalization (OR = 1.6, p = 0.053). Similarly, multivariate analysis of isolates with known site of isolation (768/795, 97%) showed a significant association between rPBP3 and eye infection (OR = 2.1, p = 0.003) but no association with other localizations. Information about STs was available for study isolates only and thus not included in the regression analysis.

The eight most prevalent STs were highly diverse with respect to resistance genotypes and clinical characteristics (Table 5). There was no correlation between rPBP3 proportions and hospitalization rates in the various STs. Three STs, two of which consisting entirely of rPBP3 isolates (ST396 and ST201) were significantly associated with eye infection (p < 0.05). ST396 was also significantly associated with the age group 0–3 yrs (p = 0.004).

### Beta-lactam susceptibility

Median MICs (MIC_50_) were generally ≥2 dilution steps higher in group II rPBP3 isolates than in sPBP3 isolates (Table 6). The single group III high-rPBP3 isolate had MICs ≥2 steps higher than MIC_50_ in group II isolates. MIC_50_ for cefotaxime differed slightly between isolates with PBP3 types A (0.03 mg/L), B (0.016 mg/L) and D (0.06 mg/L). There were otherwise no significant differences (within ±1 dilution step) between MIC_50_ in various PBP3 types, nor between sPBP3 isolates in the two study groups.

**Table 6:** Beta-lactam susceptibility according to PBP3 resistance genotypes

**Study groups**^**a**^	**Resistance genotypes**^**b**^		***n***	**MIC**_**50**_**/MIC**_**90 **_**(mg/L) and susceptibility categorization (%)**^**c**^					
				**AMP**^**c**^	**AMC**^**c**^	**PIP**^**c**^	**CXM**	**CTX**	**MEM**
Resistant group	High-rPBP3	Group III	1	8/-	16/-	0.06/-	>16/-	0.25/-	1/-
				(0/100)	(0/100)		(0/0/100)	(0/100)	(0/100/0)
		Group III-like	2	2/4	8/16	0.06/0.12	>16/>16	0.06/0.12	0.03/0.03
				(0/100)	(0/100)		(0/0/100)	(100/0)	(100/0/0)
	Low-rPBP3	Group II	111	2/4	4/8	0.03/0.06	8/8	0.03/0.12	0.12/0.5
				(40/60)	(45/55)		(33/11/56)	(94/6)	(80/20/0)
		Group I	2	0.5/1	0.25/1	0.03/0.06	0.5/16	0.06/0.25	0.016/0.06
				(100/0)	(100/0)		(50/0/50)	(50/50)	(100/0/0)
	sPBP3		60	0.25/0.5	0.5/2	0.004/0.03	1/8	0.008/0.06	0.03/0.12
				(98/2)	(98/2)		(74/13/13)	(98/2)	(100/0/0)
Susceptible group	sPBP3		19	0.12/0.5	0.5/2	0.004/0.06	0.5/8	0.004/0.03	0.03/0.12
				(100/0)	(95/5)		(79/11/11)	(100/0)	(100/0/0)

^a^See Figure 1.

^b^See Table 1.

^c^MICs (microbroth dilution) and susceptibility categorization (S/R or S/I/R) according to EUCAST clinical breakpoints [37]. The following breakpoints were used (S≤/R>): Ampicillin (AMP), 1/1; amoxicillin (AMC), 2/2; cefuroxime (CXM), 1/2; cefotaxime (CTX), 0.12/0.12; meropenem (MEM), 0.25/1. Clinical breakpoints for piperacillin and piperacillin-tazobactam are not set by EUCAST. Meningitis breakpoints were used for categorization of meropenem.

^d^For beta-lactamase positive isolates, ampicillin, amoxicillin and piperacillin MICs were determined in combination with sulbactam (4 mg/L), clavulanate (2 mg/L) and tazobactam (4 mg/L), respectively.

The majority of group II isolates had MICs above the S-breakpoints for ampicillin, amoxicillin and cefuroxime. Significant proportions were resistant to cefotaxime (7/111, 6%) and non-susceptible to meropenem (22/111, 20%), with representatives from all four major rPBP3 strains. Notably, 12% (13/111) of group II isolates were categorized as susceptible to all agents, whereas 24% (19/80) of sPBP3 isolates were non-susceptible to ≥1 beta-lactam, most commonly intermediately susceptible to cefuroxime (*n =* 10). No association with ST or phylogroup was observed.

The prevalences of clinical PBP3-mediated resistance to ampicillin and cefotaxime and non-susceptibility to meropenem in the original population (*n =* 795) were 9%, 1.3% and 2.9%, respectively.

## Discussion

### Resistance epidemiology

We found a 15% prevalence of rPBP3 in a nationwide collection of 795 eye, ear and respiratory isolates of *H. influenzae* in Norway. The prevalence of clinical resistance to ampicillin due to rPBP3 was 9%, compared to 2.5% in a similar study three years earlier [11]. Despite methodological differences between the two studies, we conclude with a significant increase from 2004 to 2007. National phenotypic surveillance data indicate a further increase to 17% rPBP3 in respiratory isolates in 2011 [40] and a prevalence at 15% rPBP3 in invasive isolates in 2012 (*n* = 73, 77% nontypeable) [41], consistent with observations in other European countries and in Canada [2,4,12,14].

As expected, group II low-level resistant isolates predominated. Notably, group III high-rPBP3 was identified for the first time in Northern Europe. The genotypic distinction between low-level and high-level beta-lactam resistance is clinically relevant: As resistance to cefotaxime is mainly seen in high-rPBP3 [6], cefotaxime is suitable for empiric treatment of severe disease only in regions where high-rPBP3 is rare. However, 6% of group II isolates in the present study were resistant to cefotaxime and 20% were non- susceptible to meropenem in case of meningitis. These observations underline the importance of confirming susceptibility to beta-lactams in severe infections such as meningitis and septicemia.

When the prevalence of low-rPBP3 in Japanese respiratory isolates reached 17% in the mid 1990s, group III isolates increased from zero to 29% in six years [13]. This was followed by a rapid increase in group III isolates in meningitis (predominantly Hib) from zero to 70% [15]. A recent report revealed a shift from low-level to high-level resistance in respiratory tract isolates in South Korea during the last decade, with an increase in the prevalence of group III isolates from 1% to 21% in five years [16,22].

A similar development in other parts of the world would seriously compromise current empiric antibiotic therapy in severe infections. So far, single group III isolates have been reported from France [14] and Canada [3] whereas group III-like isolates are slightly more frequent [9,14,18,20,21,24]. Clusters of group III and group III-like high-level resistant isolates were recently observed in Norway (Skaare *et al.*, manuscript in preparation).

The current epidemiologic situation in Europe and Canada, with a gradually increase in low-rPBP3 and sporadic reports of high-rPBP3 isolates, strongly resembles the situation in Japan and South Korea prior to the shifts in resistance genotypes. Continuous monitoring of susceptibility to cefotaxime and meropenem is necessary to ensure safe empiric treatment.

### Molecular epidemiology

By comparing the study isolates with isolates from a comparable population collected in 2004 [11], we were able to study the clonal dynamics of PBP3-mediated resistance. The increasing prevalence of rPBP3 in Norway is due to expansion of a few clones. Four STs with characteristic *ftsI* alleles accounted for 61% of the rPBP3 isolates in the present study. Two of these strains were the main contributors to PBP3-mediated resistance in Norway three years earlier [11]. Interestingly, the replacement of ST14 by ST367 as the most prevalent rPBP3 strain did not cause a shift in PBP3 type nor phylogroup, as both STs carried PBP3 type A and belong to eBURST group 2.

We have previously suggested the existence of one or more widely disseminated rPBP3 clones [11]. This is supported by later reports of PBP3 type A and compatible substitution patterns (identical to PBP3 type A as far as comparison is possible) being common in Europe [4,18,23-25], Canada [3,12], Australia [20] and South Korea [16,22], and by the present study.

PBP3 type A is frequently linked to ST14 and ST367 in the limited number of previous reports on the molecular epidemiology of rPBP3. Studies on invasive *H. influenzae* in Canada in the periods 2000–2006 [2,12,42] and 2008–2009 [3] revealed an increasing prevalence of rPBP3 in NTHi, with PBP3 type A being common in both sampling periods [3,12]. ST14 and ST367, respectively, were the most common STs in NTHi from two different regions and sampling periods [3,42]. PBP3 type A was by far the most frequent substitution pattern in ST14 and also appeared in some ST367 isolates (R. Tsang, personal communication).

Furthermore, a study on invasive *H. influenzae* in Sweden [4] identified a cluster of seven NTHi isolates of ST14 and related STs (hereunder ST367), all carrying PBP3 type A and collected in the period 2008–2010 (F. Resman, personal communication). Finally, in two recently published Spanish studies, ST14 and/or ST367 isolates with substitution patterns compatible with PBP3 type A were reported in invasive disease (ST367, *n* = 2) [24] and pneumonia (ST14, *n* = 2; ST367, *n* = 1) [25] in the period 2000–2009.

The *ftsI* alleles encoding type A in this and our previous study [11] had high genetic similarity and alleles in separate clusters rarely encoded identical PBP3 types. Thus, despite the lack of cross-study comparison of *ftsI* DNA sequences, the examples above indicate that clonal distribution is a more likely explanation for the occurrence of PBP3 type A and compatible patterns in separate studies from four continents [3,4,9,11,12,16,18,20],[22-25] than independent development of this substitution pattern by convergence.

Importantly, an invasive high-level resistant rPBP3 isolate with the same combination of MLST allelic profile (ST155) and PBP3 substitution pattern as the two group III-like isolates in the present study was recently reported from Spain [24]. A single-locus variant (ST1118) with an identical substitution pattern was also reported.

These observations are notable and support the need of global surveillance initiatives. We here show that combining MLST and PBP3 typing provides a tool for cross-study identification of rPBP3 strains and clones. The previously suggested system for subgrouping of group II isolates [38] does not separate PBP3 types [11,16] and is unsuitable for this purpose.

Preferably, MLST should be combined with *ftsI* DNA sequencing. The *ftsI* gene is nearly 200 kb from its nearest MLST neighbor (*mdh*) and distortion of the MLST results due to linkage is thus very unlikely. With recent technological development reducing both costs and analysis time of whole-genome sequencing, and smaller bench-top sequencers becoming readily available, MLST-*ftsI* typing will probably be possible to perform for surveillance purposes in the near future.

We are aware of a number of previous studies where MLST and *ftsI* sequencing was performed [3,4,12,23-25,43-45]. To our knowledge, four reports have linked MLST data and PBP3 substitution patterns: one presented the allelic profiles of 83 group III respiratory isolates from Japan [43]; another presented the substitution pattern of a single group II ST368 NTHi isolate causing meningitis in Italy [44]; and two most recent publications presented the substitution patterns and STs of 95 respiratory [25] and 18 invasive isolates [24] from Spain. However, the present study is to our knowledge the first to connect STs to *ftsI* alleles.

PFGE is highly discriminative and generally considered suited for assessment of relatedness between epidemiologically connected isolates, particularly in populations with high recombination rates such as NTHi [39,46]. In this study, PFGE clusters correlated well to MLST clonal complexes. Band patterns were stable over time and also traced phylogenetic relationship not detected by MLST and parsimony analysis. Combining MLST and PFGE for typing of NTHi may thus increase both sensitivity and resolution of clone detection.

### Development of resistance

As discussed above, clonal expansion is important for the spread of rPBP3. However, the PBP3 type A-encoding, highly divergent *ftsI* allele *lambda-2* was distributed among several unrelated STs. Similar observations are reported previously but only in studies using PFGE for strain characterization [11,26]. Exchange of complete alleles by HGT seems the most likely explanation, and has been demonstrated in vitro [26]. The mechanisms for HGT of *ftsI* sequences in *H. influenzae* are not completely resolved but involvement of classical transformation and homologous recombination has been suggested [26,47].

Transformational competence varies extensively between *H. influenzae* strains [48]. This implies that the ability to acquire mutant *ftsI* alleles encoding rPBP3 will vary correspondingly, which may explain the differences in ST and phylogroup distribution between rPBP3 and sPBP3 isolates. It has been suggested that phylogroups are maintained by restriction barriers, preventing recombination between isolates of different heritage [32]. This is challenged by the distribution of *lambda-2* to several phylogroups. A simple explanation may be that restriction barriers prevent recombination between some phylogroups and allow recombination between others.

Recent studies applying whole-genome sequencing have revealed that transformation in competent strains of *H. influenzae* is more extensive than previously recognized [49] and that transformational exchange may cause allelic variation involving complete genes between strains of identical STs [50]. However, transfer of complete *ftsI* alleles is probably less common than exchange of shorter sequences, causing mosaicism [26,28]. Preliminary multiple sequence alignment analysis of *ftsI* sequences in this study indicated intrageneic recombination (data not shown).

### PBP3-mediated resistance and virulence

The association between rPBP3 and virulence is poorly described. One experimental study reported increased ability of a group III NTHi strain to invade bronchial epithelial cells, and the authors hypothesized that rPBP3 may enhance virulence by acting as an adhesion molecule [51]. A more recent retrospective epidemiological study concluded with no difference in pathogenicity between rPBP3 and sPBP3, but an association between rPBP3 and underlying respiratory disease was observed [17]. Molecular strain characterization was not performed in any of the two studies.

In the present study, regression analysis (without adjustment for ST) suggested that rPBP3 is associated with increased risk of eye infection and hospitalization. However, ST-specific analysis indicated that pathogenicity is correlated with STs rather than with resistance genotypes. For instance, ST395, ST396 and ST201 were significantly associated with eye infections but only the two latter STs were associated with PBP3-mediated resistance.

Irrespective of resistance genotypes, a number of STs in this study had clinical characteristics recognizable from previous reports: ST159 was predominantly isolated from the respiratory tract of hospitalized elderly patients, consistent with ST159 being adapted to infection in chronic obstructive pulmonary disease (COPD) [52]; and the high proportion of ST57 in children is consistent with a previously reported association with acute otitis media (AOM) [53]. Finally, the high hospitalization rate of patients with ST14-PBP3 type A corresponds well with the potential of this strain to cause pneumonia [25] and invasive disease [3,4,42].

These observations are in accordance with a recent population study suggesting association between population structure and disease [53]. In conclusion, the association between rPBP3 and pathogenicity suggested by the regression analysis most likely reflects that some of the most frequently occurring rPBP3 strains in this study also possessed strain-associated virulence properties. Identification of virulence determinants is beyond the scope of this study. However, our observations underline that studies on the correlation between resistance genotypes and pathogenicity should include molecular strain characterization. Accordingly, the previously reported association between PBP3-mediated resistance and clinical characteristics [17,51] may be spurious.

## Conclusions

The prevalence of rPBP3 in *H. influenzae* is increasing worldwide, and high-level resistant strains are emerging in new geographic regions.

In this study of eye, ear and respiratory isolates in Norway, the rPBP3 prevalence was 15%, with four strains accounting for 61% of the resistant isolates. Group II low-rPBP3 isolates predominated, and significant proportions of isolates were non-susceptible to cefotaxime and meropenem. Group III high-rPBP3 was identified for the first time in Northern Europe.

The results support a role of horizontal gene transfer in the emergence of rPBP3 and indicate phylogeny restricted transformation. Comparative analysis with data from previous studies indicates wide dissemination of clonally related rPBP3 strains. Notably, two strains highly prevalent in Norway (ST14 and ST367 with PBP3 type A) are common in invasive disease in Europe and Canada.

Continuous monitoring of beta-lactam susceptibility is necessary to ensure safe empiric therapy in severe disease and to detect a future shift from low-level to high-level resistance. The need of a global system for molecular surveillance of rPBP3 strains is underlined. The novel approach of combining MLST and *ftsI*/PBP3 typing is a powerful tool for this purpose.

## Abbreviations

NTHi: Nontypeable *Haemophilus influenzae*; Hib: *Haemophilus influenzae* serotype b; Hif: *Haemophilus influenzae* serotype f; PBP3: Penicillin-binding protein 3; BLNAR: Beta-lactamase negative ampicillin resistant; BLPACR: Beta-lactamase positive amoxicillin-clavulanate resistant; rPBP3: PBP3-mediated resistance present (‘resistant’ PBP3); sPBP3: PBP3-mediated resistance absent (‘susceptible’ PBP3); MLST: Multilocus sequence typing; ST: Sequence type; CC: Clonal complex; Phylogroup: Phylogenetic group; PFGE: Pulsed-field gel electrophoresis; HGT: Horizontal gene transfer; UPGMA: Unweighted pair group method with arithmetic mean; EUCAST: European Committee on Antimicrobial Susceptibility Testing; MIC: Minimal inhibitory concentration; COPD: Chronic obstructive pulmonary disease; AOM: Acute otitis media; NORM: Norwegian Surveillance Programme for Antimicrobial Resistance; R-group: Resistant group; S-group: Susceptible group.

## Competing interests

The authors declare that they have no competing interests.

## Authors’ contributions

DS conceived and coordinated the study, performed susceptibility testing, analysed and interpreted data and wrote the first draft; BEK, YT, AJ, LS and AS contributed to study design; ILA designed and undertook molecular analyses (except MLST); LS analysed the PFGE data, DAC and MS were responsible for acquisition of MLST data and AJ advised on bioinformatics. All authors participated in interpretation of results, critically revised the draft for intellectual content and approved the final article.
